# Proceedings of the Global Health Focus Youth Conference 2025: The Role of Young People in Advancing Global Health Education, Research, and Leadership

**DOI:** 10.1002/puh2.70346

**Published:** 2026-08-13

**Authors:** Zhinya Kawa Othman, Don Eliseo Lucero‐Prisno, Mohamed Mustaf Ahmed, Olalekan John Okesanya, Aniekan Michael Ekpenyong, Jerico Bautista Ogaya, Goodness Ogeyi Odey, Adriana Viola Miranda, Yasir Ahmed Mohammed Elhadi, Yusuff Adebayo Adebisi

**Affiliations:** ^1^ Department of Pharmacy Kurdistan Technical Institute Sulaymaniyah Kurdistan Region Iraq; ^2^ Department of Global Health and Development London School of Hygiene and Tropical Medicine London UK; ^3^ Research and Development Office Guimaras State University Guimaras Philippines; ^4^ Research and Development Office Palompon Institute of Technology Palompon Philippines; ^5^ Faculty of Medicine and Health Sciences SIMAD University Mogadishu Somalia; ^6^ Faculty of Medicine, Department of Public Health and Maritime Transport University of Thessaly Volos Greece; ^7^ Department of Research Global Health Focus Africa Abuja Nigeria; ^8^ Department of Medical Technology, Institutions of Health Sciences and Nursing Far Eastern University Manila Philippines; ^9^ Center for University Research University of Makati Makati Philippines; ^10^ Department of Public Health and Health Policy London School of Hygiene and Tropical Medicine London UK; ^11^ Department of Health Policy London School of Economics and Political Science London UK; ^12^ Department of Research Global Health Focus Asia Bandung Indonesia; ^13^ Department of Public Health Institute, College of Medicine and Health Sciences United Arab Emirates University Al Ain United Arab Emirates; ^14^ College of Social Sciences University of Glasgow Glasgow UK

**Keywords:** education, equity, global health, leadership, research, youth

## Abstract

Youth represent the largest and most interconnected generation but remain underserved by traditional and global health structures. In Africa alone, nearly 60% of the population is under the age of 25, and the continent's youthful population is projected to reach almost 1 billion by 2050. This narrative synthesis draws on proceedings from the 2025 Global Health Focus Youth Conference in Kigali, Rwanda, and triangulates them with recent peer‐reviewed evidence to discuss how young professionals contribute to health equity in three domains: education, research, and leadership. Participants called for mandatory equity‐oriented curricula grounded in local realities, expanded mentorship networks, and scholarship schemes that lower barriers for students from low‐ and middle‐income countries (LMICs). Research data reveal persistent authorship and funding asymmetries, but targeted seed grants and South‐led capacity‐building programs are increasing local first‐ and last‐author representation. Conference dialogues underscored that equitable partnerships, reinforced by funder requirements and journal policies, are key to decolonizing knowledge production. Leadership discussions have highlighted successful youth‐driven innovations and advocacy campaigns that influence national and global agendas. Synthesizing these insights, this article offers recommendations for universities, funders, and policymakers to institutionalize mentorship, earmark youth‐led research funds, and legislate youth seats in governance. Implementing these actions would transform young people from occasional consultees into co‐architects of sustainable, context‐responsive global health solutions.

## 1 Introduction

The current global health landscape is defined by a complex and turbulent environment marked by profound and often converging crises and long‐term trends that significantly compromise human health and well‐being. Increasing inequities both within and between countries, exacerbated by the COVID‐19 pandemic, have created a growing divide in health outcomes, with approximately 2.2 billion people (around a quarter of the global population) lacking access to safely managed drinking water [1] and one in four people suffering financial hardship in accessing health services [2]. Global commitments, such as the 2018 Astana Declaration, enshrined the goal of universal life‐course primary health care (PHC) with community engagement and multisectoral action. However, in most low‐ and middle‐income countries (LMICs), public funding for PHC remains inadequate, access is inequitable, and out‐of‐pocket payments are common [3]. Additionally, the backdrop is a constant and growing number of crises and emergencies, with a record 339 million people requiring humanitarian assistance in 2023, coupled with economic uncertainty, including slow health system recovery, rising debt burdens, and shrinking fiscal space [4]. This tumultuous context has had an unacceptable impact on human health and well‐being, as evidenced by less than 15% of health‐related Sustainable Development Goals (SDGs) being on track and a slowing rate of healthy life expectancy improvement since 2015 [2]. Addressing these gaps requires promoting local innovation and youth engagement in shaping a global health agenda and advocating healthier choices and environments. In this context, the Global Health Focus (GHF) Youth Conference 2025, convened in Kigali, Rwanda, provided a timely platform to examine how young people can contribute to equity‐oriented reforms in global health policy.

The global youth population stands at 1.2 billion (16% of the world's population); however, their potential to influence health agendas remains underutilized. In Africa, where nearly 60% of the population is under 25 years of age and youth are projected to reach almost 1 billion by 2050, accounting for over 40% of the global youth population by 2030, their demographic significance highlights the urgent need to prioritize health [5]. In sub‐Saharan Africa (SSA), 70% of inhabitants are under 30 years of age, creating an unprecedented demographic dividend that still lacks commensurate investment in health leadership [6]. In the Middle East and North Africa (MENA) region, approximately 110 million youth aged 15–29 live in the Arab states, accounting for approximately 30% of the region's population, and over 60% of the population is under 30 years of age [7]. Although the World Health Organization (WHO) now embeds youth in advisory structures, such as the WHO Youth Council, tangible decision‐making power for young professionals in education, research, and policy, is sporadic [8].

According to United Nations (UN) classifications, youth are commonly defined as individuals aged 15–24 years. Despite their demographic significance, gaps remain in the fields of education, research, and leadership. In education, tertiary enrolment in sub‐Saharan Africa remains approximately 9%, far below the global average of nearly 39%, limiting the pipeline of future global health professionals [9, 10]. In research, the African region accounts for only about 1.1% of global R&D investment and roughly 2% of world research output despite comprising 15% of the world's population, contributing to inequities in research output and leadership opportunities for young scientists [11]. In leadership, youth participation in formal decision‐making structures remains limited, with global indices indicating lower participation scores in SSA compared to global averages, reflecting persistent structural barriers to meaningful youth representation in health governance [12]. Bibliometric evidence shows that in cancer research supported by the US National Cancer Institute involving LMICs, only approximately 22% of publications had a first author affiliated with an LMIC, and 51% had no LMIC‐affiliated author at all, signaling persistent authorship inequities [13]. Closing this evidence gap is important because equitable authorship, participatory training models, and youth‐driven innovation are recognized levers for achieving the SDGs and Universal Health Coverage (UHC). This article aims to synthesize insights from the GHF Youth Conference 2025 and relevant literature to examine youth contributions to global health education, research, and leadership and proposes actionable recommendations for academia, governments, and development partners.

## 2 Conference Overview and Methodological Approach

The GHF Youth Conference 2025, held in Kigali, Rwanda, served as a dedicated forum to elevate youth engagement in global health. Organized as part of GHF's 10th anniversary celebrations on July 18, 2025, the event marked a decade of empowering young leaders from over 30 countries across Africa, the Middle East, Europe, and Asia. Approximately 100 participants attended the conference in person, whereas an additional 20 young people joined virtually through live streaming platforms, reflecting the forum's commitment to inclusivity and accessibility. The conference aimed to highlight the pivotal role of young people in shaping the future of global health, particularly in the post‐pandemic era, where innovation and resilience are crucial. Unlike traditional global health meetings, this youth‐centered conference provided a platform for emerging researchers, educators, and advocates to share their perspectives on health equity. By focusing on transformative education, equitable research partnerships, leadership development, and youth‐driven innovation, the forum's unique positioning aligns young people's energy and ideas with global health priorities. Additionally, it responds to the growing recognition that meaningful youth involvement is essential for achieving sustainable health outcomes and justice worldwide.

The single‐day program included seven substantive sessions delivered in a single‐track format, organized around three core thematic tracks: education and research, leadership and innovation, and youth empowerment for health equity. The sessions included two keynote addresses, two panel discussions, two interactive sessions, a collaborative dialogue, and abstract presentations by participants. No parallel workshops or breakout sessions were conducted. Key points from each session were documented through structured note‐taking by the organizing team and subsequently reviewed and thematically organized by the authors to inform the present synthesis of the findings. This article is a narrative synthesis of conference proceedings triangulated with peer‐reviewed literature, where insights from the keynote address, panel discussions, interactive sessions, abstract presentations, and collaborative dialogue were thematically analyzed and integrated with published evidence to contextualize emerging priorities in global health education, research, leadership, and innovation.

The following sections synthesize the key conference themes and examine their implications for practice and policy. Insights identified as arising from conference discussions reflect participant perspectives, whereas the supporting literature provides empirical grounding, and the authors’ interpretations synthesize both sources to propose actionable recommendations. This conference summary documents actionable strategies developed by youth leaders to tackle health disparities, advance research equity, and influence policy and recommends pathways for integrating youth‐driven solutions into future global health initiatives. Consistent with the conference mandate, we emphasize how youth‐focused education, research, leadership, and innovation can create lasting improvements in health systems and their outcomes.

## 3 Global Health Equity

Global health is a multidisciplinary field that prioritizes worldwide health equity by addressing transnational determinants through collaboration across health and non‐health disciplines, integrating population‐level prevention with individual clinical care, and, in its modern framing, explicitly incorporating environmental and climate change concerns [14]. Severe inequities persist in global health, exemplified by the COVID‐19 vaccine rollout, where affluent nations used their political and financial clout to secure more than twice the doses needed for their populations, whereas low‐income countries (LICs), lacking comparable leverage, had vaccination rates that remained below 1% in early 2021, a disparity often described as “vaccine apartheid” [15]. Furthermore, a new WHO global report on social determinants of health equity emphasizes that factors such as inadequate housing, limited education, and poor job prospects drive substantial and often decades‐long reductions in healthy life expectancy, with gaps of up to 33 years between countries, underscoring that these social determinants outweigh genetic influences or even access to medical care in shaping health outcomes [16].

These inequities are also reflected in disparities in access to training opportunities, influence over knowledge production, and representation in decision‐making structures within the global health systems [17]. In education, access to advanced training remains uneven, with the gross tertiary enrolment ratio in sub‐Saharan Africa standing at only around 9%, well below the global average of roughly 40%, highlighting inequities in the global health talent pipeline [18, 19]. In research, research output remains highly skewed, with African researchers contributing only approximately 3% of global research output, reflecting persistent underinvestment in research capacity that limits opportunities for locally driven scientific leadership [20]. In leadership and partnerships, analyses of global health consortia indicate that institutions from high‐income countries lead most multicountry health projects, whereas LMIC partners frequently serve implementation roles, reflecting continuing asymmetries in agenda‐setting authority and decision‐making power in global health governance structures [21].

Thus, global health equity demands systemic changes in education, research, and partnerships. This aligns with transformative education theory, which argues that meaningful learning goes beyond knowledge transfer to critical reflection on assumptions, power relations, and social structures, thereby enabling learners to become agents of change. In global health, this perspective supports curricula that center on the local context, challenge inequitable norms in knowledge production, and equip learners to translate reflection into equity‐oriented action [22, 23]. Applied to global health training, this approach supports curricula that strengthen context‐sensitive problem‐solving, promote equitable research collaboration, and build competencies for ethical leadership and policy engagement in diverse settings [24]. Universities worldwide are increasingly urged to embed equity‐focused curricula that prepare students to tackle local health disparities, emphasizing that new global health training must integrate local contexts and values [25]. From our first panel discussion, the assertion was made that “educators stress the need to retain our own ways of thinking while critically adopting global health methodologies that reckon with our local contexts to advance health education.” Simultaneously, young researchers, especially in LMICs, face persistent inequities in funding and publishing opportunities, reflecting the under‐representation of LMIC investigators in leading research roles [13]. Such disparities in knowledge production and authorship fuel calls to decolonize global health by promoting equitable research partnerships and shifting decision‐making power toward local experts. Panelists further noted that meaningful progress in global health equity requires strengthening pathways that enable youth to contribute to locally relevant solutions through training, mentorship, and research. Examples shared during the Rwanda conference illustrated how youth‐led inquiry can address community‐specific health priorities, demonstrating the potential of locally driven innovation while acknowledging the need for continued evaluation of the long‐term impact.

## 4 Youth Leadership and Innovation in Global Health

The Youth2030 UN Youth Strategy positions young people as essential drivers of sustainable, inclusive, and peaceful societies, aiming to secure their human rights, maximize their potential, and embed their voices in policymaking through a coherent, accountable, and transparent framework to achieve SDGs [26]. Strengthening youth leadership has implications beyond representation alone, as evidence suggests that youth engagement can influence policy priorities, improve the uptake of health interventions through peer‐driven communication, and strengthen accountability mechanisms by ensuring that policies reflect the lived realities of affected populations. Thus, youth leadership contributes to more responsive, equitable, and sustainable global health systems [27, 28]. Aware that tomorrow is theirs to shape, young people are primed to drive progress toward a fair, inclusive, and efficient world. They currently constitute the planet's largest age cohort, with half of the population being under 20 years of age in the least developed countries, and the population aged 12–17 years is projected to increase from 153 million in 2023 to 223 million by 2050 [29].

The sheer number of young people makes them an important stakeholder in pushing for global health equity. Furthermore, young people possess important qualities necessary for promoting global development, and their energy, ideals, and imagination have been recognized by stakeholders, such as the UN. Their curious mindset and appetite for collaboration also uniquely position them as inclusive changemakers. Simultaneously, youth are not a homogeneous group, and opportunities for participation are shaped by intersecting social positions. Gender, disability, socioeconomic background, and geographic or regional location can strongly influence who is heard, who can access leadership pathways, and who benefits from youth‐focused policies [28, 30]. Despite this potential, early career professionals often lack access to mentorship, role models, and leadership training that would enable them to translate their energy and ideas into policy influence. Indeed, meaningful youth engagement is still emerging among the 194 WHO member states; only 13 countries (about 6.7%) included youth delegates at the 2023 World Health Assembly (an increase from just nine countries, 4.6%, in 2022), and 11 of those 13 were high‐income countries [31]. Importantly, participation does not necessarily equate to decision‐making authority, as youth representatives are frequently included in advisory capacities without voting rights or formal agenda‐setting power within national or global health governance bodies, illustrating the gap between symbolic inclusion and institutional influence [32, 33].

Such statistics highlight persistent barriers, ranging from limited youth representation in governance to systemic exclusion of youth. Notably, one scoping review found that fewer than 1% of youth‐focused health studies that collected data from young people involved them in a genuine advisory role, underscoring the urgent need to address these gaps to fully harness the potential of youth leadership [34]. A recent evidence synthesis found that young experts face challenges at multiple levels, including a lack of institutional support, tokenistic inclusion, where youth are formally included but their voices carry little real influence, and discrimination. These challenges are often intensified for young women, youth with disabilities, and those from lower‐income or marginalized communities, who may face additional obstacles related to mobility, digital access, affordability, stigma, and institutional bias [28, 30]. Taken together, these layered inequities hinder meaningful participation and underscore the need for deliberate mentorship opportunities and inclusive policies to empower young leaders [28]. Therefore, an intersectional approach is essential. Youth engagement mechanisms should be designed to ensure representation across gender, disability status, and socioeconomic groups, rather than relying on one‐size‐fits‐all participation models [28, 30]. Despite these obstacles, youth‐driven innovation is already contributing to transformative solutions in global health. Globally, young innovators are leveraging community engagement and digital technology to improve health outcomes. For example, a 2022 global crowdsourcing call identified 99 youth‐led health initiatives (from LMICs) and found that over 60% of these innovations had already been piloted or implemented in their communities. Many of these youth‐led solutions are low‐cost, contextually tailored, and sustainability‐oriented, aligning with public health needs in resource‐limited settings [35]. However, relatively few youth‐led initiatives serve as primary recipients of large‐scale global health funding, highlighting ongoing structural constraints in leadership authority despite demonstrated innovative capacity [36].

From the second panel discussion, the importance of translating academic research into real‐world impacts through innovation was a recurring theme. Young leaders are encouraged to critically assess their skills, build supportive networks, and align their projects with community needs and government priorities to ensure that their ideas are both feasible and scalable. Speakers emphasized that mentorship and collaboration are key; connecting youth with institutions and policymakers helps bridge the gap between research and practice, and peer networks enable horizon scanning for emerging health challenges and technological tools. Several case studies have highlighted how early mentorship and partnerships with stakeholders can amplify youth‐led projects. For instance, youth innovators who received mentorship and engaged local communities co‐developed digital health solutions tailored to underserved populations in LMICs, with evidence of their feasibility, uptake, and implementation potential [35, 37]. These examples show that positioning youth as co‐creators rather than passive participants enhances the relevance, feasibility, and sustainability of global health innovation. Meaningful youth leadership, therefore, represents not symbolic inclusion, but a strategic approach to strengthening responsiveness, accountability, and long‐term impact in global health systems.

## 5 Call to Action

**FIGURE 1:**
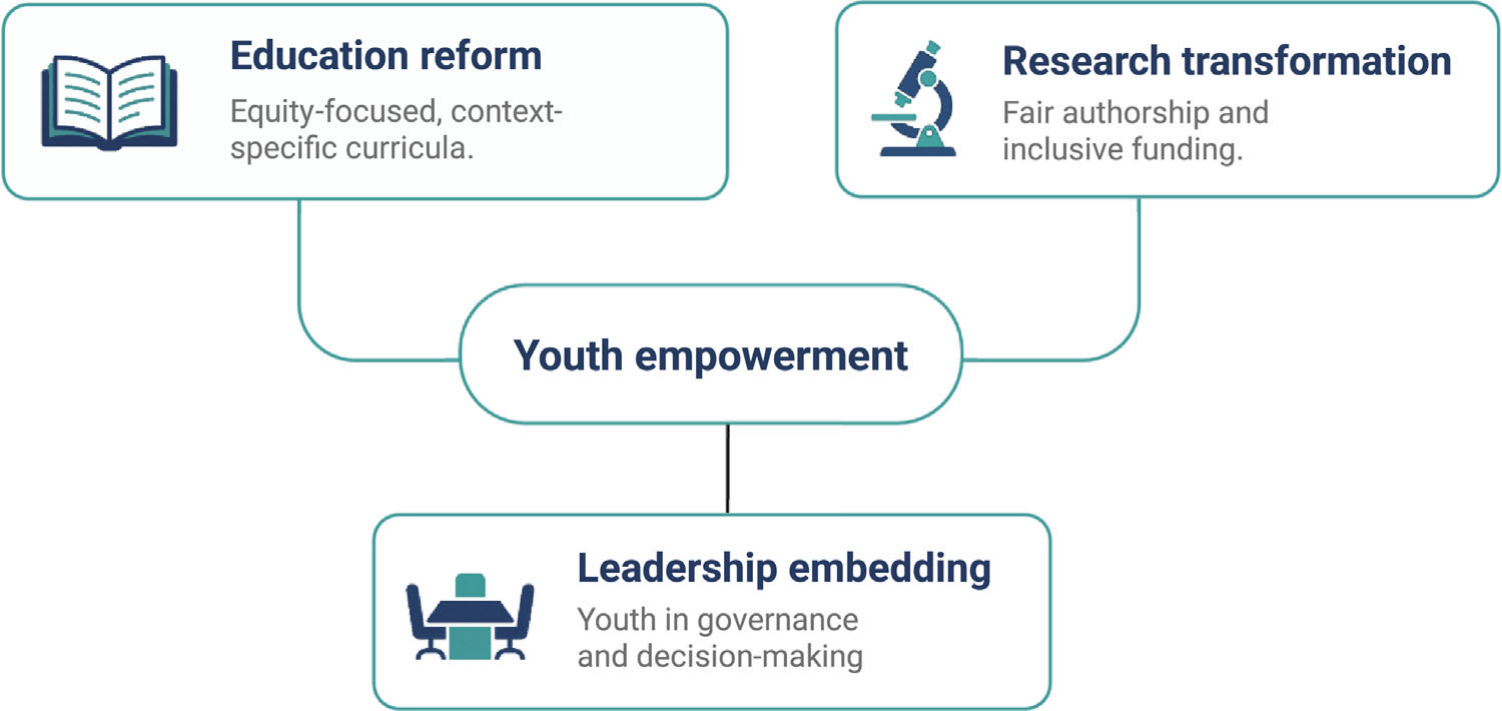
Youth empowerment pillars for sustainable global health for all.

To accelerate the contribution of young people to global health, academic institutions, policymakers, funders, and youth networks must pursue a three‐pronged agenda that simultaneously reforms education, transforms research structures, and embeds youth leadership at every governance level, while foregrounding mentorship and equity, particularly for those in fragile states (Figure 1). First, universities should embed equity‐focused, context‐specific content across medical and public health curricula, because evidence shows that even in high‐income settings, only 79% of global health learning outcomes are systematically covered, whereas surveys in Sudan and the United Arab Emirates reveal large knowledge gaps among students when global health is treated as an elective [38, 39, 40]. National regulators should therefore mandate core global health modules and create scholarship schemes to widen access for underrepresented youth, whereas funders should tie grants to demonstrable curriculum reform and formal mentorship arrangements that pair early career trainees with experienced faculty. Second, research ecosystems must dismantle authorship and funding asymmetries by reserving targeted seed grants and leadership slots for youth investigators, as only one‐third of first authors and roughly one‐fifth of senior authors in papers from LICs are locally affiliated [41], and a mere 0.2 % of major global health research grants flow directly to institutions in those settings [42]. Thus, funders should make equitable authorship a reporting requirement and expand South‐led, youth‐directed grant programs, whereas journals should prioritize reviews that reward local leadership. Third, leadership pipelines must be institutionalized by embedding youth seats in global and national decision‐making forums and funding structured mentorship at scale, as the lack of mentors remains a chief obstacle to diverse global health careers [43]. Governments should legislate youth quotas on advisory boards and recognize youth‐led innovations through public procurement or matched funding schemes. Lastly, all stakeholders must address the investment gap, as channeling resources into youth‐designed digital health and community enterprises proves highly effective, with over 60% of such initiatives reaching implementation even with modest funding [35]. Taken together, these evidence‐based recommendations would convert the energy and creativity showcased at the 2025 GHF Youth Conference into sustained structural change, positioning young professionals not as periodic consultees but as co‐architects of a more equitable global‐health future [44].

## 6 Conclusion

Global health, the pursuit of equitable health and well‐being for all people across borders, is foundational to social stability, economic resilience, and collective security. The evidence reviewed here demonstrates that when universities embed locally relevant, equity‐focused curricula, funders allocate grants that place young LMIC investigators in lead roles, and decision‐making bodies reserve formal seats for youth, measurable gains follow, ranging from increased local authorship to scalable digital health innovations. Persistent gaps in mentorship, funding, and representation reveal that global health institutions are still organized around hierarchies that marginalize young voices, particularly from resource‐limited regions. By operationalizing the recommendations distilled from the conference, stakeholders can convert the passion and creativity of today's young professionals into sustained structural change, ensuring that the next decade of global health is shaped and led by those whose futures depend on it.

## Author Contributions

Zhinya Kawa Othman and Don Eliseo Lucero‐Prisno conceptualized and designed the study. Zhinya Kawa Othman and Olalekan John Okesanya performed the literature review and data curation. Zhinya Kawa Othman and Mohamed Mustaf Ahmed drafted the manuscript. Don Eliseo Lucero‐Prisno supervised the study. Aniekan Michael Ekpenyong, Jerico Bautista Ogaya, Goodness Ogeyi Odey, Adriana Viola Miranda, Yasir Ahmed Mohammed Elhadi, and Yusuff Adebayo Adebisi contributed critical intellectual input. And all authors have read and approved the final manuscript.

## Funding

The authors have nothing to report.

## Ethics Statement

The authors have nothing to report.

## Consent

The authors have nothing to report.

## Conflicts of Interest

The authors declare no conflicts of interest.

## Data Availability

Not applicable; No new datasets were generated or analyzed.

## References

[1] UNICEF , Progress on Household Drinking Water, Sanitation and Hygiene 2000–2022: Special Focus on Gender (UNICEF, 2023), https://data.unicef.org/resources/jmp‐report‐2023/.

[2] WHO , A Global Health Strategy for 2025–2028 (WHO, 2025), https://cdn.who.int/media/docs/default‐source/documents/about‐us/general‐programme‐of‐work/global‐health‐strategy‐2025‐2028.pdf.

[3] K. Hanson , N. Brikci , D. Erlangga , et al., “The Lancet Global Health Commission on Financing Primary Health Care: Putting People at the Centre,” Lancet Global Health 10, no. 5 (2022): e715–e772, 10.1016/S2214-109X(22)00005-5. 35390342 PMC9005653

[4] OCHA , Global Humanitarian Overview 2023 (OCHA, 2023), https://www.unocha.org/publications/report/world/global‐humanitarian‐overview‐2023‐enaresfr.

[5] United Nations Youth , Definition of Youth (United Nations Youth, 2008), https://www.un.org/esa/socdev/documents/youth/fact‐sheets/youth‐definition.pdf.

[6] United Nations , Young People's Potential, the Key to Africa's Sustainable Development (United Nations, 2021), https://www.un.org/ohrlls/news/young‐people%E2%80%99s‐potential‐key‐africa%E2%80%99s‐sustainable‐development.

[7] UNFPA , Adolescents and Youth in the Arab States Region (UNFPA, 2026), https://arabstates.unfpa.org/en/topics/youth‐participation‐leadership.

[8] WHO , WHO Youth Engagement (WHO, 2026), https://www.who.int/initiatives/who‐youth‐engagement.

[9] World Bank Group , School Enrollment, Tertiary (% Gross)—Sub‐Saharan Africa (World Bank Group, 2026), https://data.worldbank.org/indicator/SE.TER.ENRR?locations=ZG.

[10] “Monitoring SDG 4: Higher Education,” UNESCO, accessed July 24, 2026, https://www.unesco.org/gem‐report/en/higher‐education.

[11] V. Simpkin , E. Namubiru‐Mwaura , L. Clarke , and E. Mossialos , “Investing in Health R&D: Where We Are, What Limits Us, and How to Make Progress in Africa,” BMJ Global Health 4, no. 2 (2019): e001047, 10.1136/bmjgh-2018-001047. PMC6407556 30899571

[12] IDD Brussels , GYPI: Data‐Driven Insights on Youth Participation (IDD Brussels, 2025), https://idd‐brussels.eu/gypi‐data‐driven‐insights‐on‐youth‐participation/.

[13] L. Eldridge , E. M. Garton , K. Duncan , and S. Gopal , “Authorship of Publications Supported by NCI‐Funded Grants Involving Low‐ and Middle‐Income Countries,” JAMA Network Open 7, no. 3 (2024): e243215, 10.1001/jamanetworkopen.2024.3215. 38551565 PMC10980966

[14] E. August , L. Tadesse , M. S. O'Neill , et al., “What Is Global Health Equity? A Proposed Definition,” Annals of Global Health 88, no. 1 (2022): 50, 10.5334/aogh.3754. 35860038 PMC9266830

[15] S. Harman , P. Erfani , T. Goronga , J. Hickel , M. Morse , and E. T. Richardson , “Global Vaccine Equity Demands Reparative Justice ‐ not Charity,” BMJ Global Health 6, no. 6 (2021): e006504, 10.1136/bmjgh-2021-006504. PMC8215249 34919057

[16] WHO , Health Inequities Are Shortening Lives by Decades (WHO, 2025), https://www.who.int/news/item/06‐05‐2025‐health‐inequities‐are‐shortening‐lives‐by‐decades.

[17] Y. H. Abdi , M. S. Abdi , S. G. Bashir , N. I. Ahmed , and Y. B. Abdullahi , “Understanding Global Health Inequality and Inequity: Causes, Consequences, and the Path Toward Justice in Healthcare,” Public Health Challenges 4, no. 4 (2025): e70156, 10.1002/puh2.70156. 41146749 PMC12553980

[18] UNESCO , Education Transforms Lives (UNESCO, 2026), https://www.unesco.org/en/education.

[19] ONE , Access to Education: Substantial, but Uneven Progress (ONE, 2020), https://www.one.org/stories/uneven‐progress‐education‐access/.

[20] J. Nabyonga‐Orem , J. A. Asamani , and O. Olushayo , “Why Are African Researchers Left Behind in Global Scientific Publications?—A Viewpoint,” International Journal of Health Policy and Management 13 (2024): 8149, 10.34172/ijhpm.2024.8149. 39099524 PMC11608291

[21] R. Ingenhoff , M. Sarker , K. Hanson , et al., “Stronger Together: Advancing Equity in Global Health Research Partnerships,” BMJ Global Health 10, no. 6 (2025): e019601, 10.1136/bmjgh-2025-019601. PMC12186048 40550574

[22] J. Mezirow , “On Critical Reflection,” Adult Education Quarterly (American Association for Adult and Continuing Education) 48, no. 3 (1998): 185–198, 10.1177/074171369804800305.

[23] M. W. Gichane and D. D. Wallace , “Dismantling and Reimagining Global Health Education,” Global Health Action 15, no. 1 (2022): 2131967, 10.1080/16549716.2022.2131967. 36285634 PMC9621231

[24] M. J. Schleiff , P. M. Mburugu , J. Cape , et al., “Training Curriculum, Skills, and Competencies for Global Health Leaders: Good Practices and Lessons Learned,” Annals of Global Health 87, no. 1 (2021): 64, 10.5334/aogh.3212. 34307067 PMC8284497

[25] K. H. Jacobsen and C. E. Waggett , “Global Health Education for the Post‐Pandemic Years: Parity, People, Planet, Priorities, and Practices,” Global Health Research and Policy 7, no. 1 (2022): 1, 10.1186/s41256-021-00234-y. 34980272 PMC8724002

[26] United Nations , About Youth2030, the UN Youth Strategy (United Nations, 2026), https://www.un.org/youthaffairs/en/youth2030/about.

[27] K. R. B. Calma , L. J. Brown , G. Fernando , and L.‐A. Omam , “Strengthening Primary Health Care: Contributions of Young Professional‐Led Communities of Practice,” Primary Health Care Research & Development 23, no. e13 (2022): e13, 10.1017/S1463423621000815. 35234118 PMC8919183

[28] K. Bailey , B. Allemang , A. Vandermorris , et al., “Benefits, Barriers and Recommendations for Youth Engagement in Health Research: Combining Evidence‐Based and Youth Perspectives,” Research Involvement and Engagement 10, no. 1 (2024): 92, 10.1186/s40900-024-00607-w. 39223602 PMC11370084

[29] United Nations , World Population Ageing 2023 (United Nations, 2023), https://www.un.org/development/desa/pd/sites/www.un.org.development.desa.pd/files/undesa_pd_2024_wpa2023‐report.pdf.

[30] C. I. Agu , O. Nwankpa , C. N. Ekwueme , et al., “Intersectionality Analysis of Young People's Experiences and Perceptions of Discrimination in Primary Health Centers in Ebonyi State, Southeast Nigeria,” International Journal for Equity in Health 23, no. 1 (2024): 100, 10.1186/s12939-024-02192-6. 38760811 PMC11100186

[31] B. O'Sullivan , A. Zhong , L. L. Yin , et al., “The Future of Global Health: Restructuring Governance Through Inclusive Youth Leadership,” BMJ Global Health 8, no. 11 (2023): e013653, 10.1136/bmjgh-2023-013653. PMC10632807 37935521

[32] T. S. Marah , H. D. Pradhan , and F. A. Shuhood , “Youth Participation in Global Governance: Opportunities and Challenges,” JoGaPA 2, no. 1 (2024): 238–249, 10.70248/jogapa.v2i1.1718.

[33] T. Holetzek and C. Holmberg , “Representation in Participatory Health Care Decision‐Making: Reflections on an Application‐Oriented Model,” Health Expectations 25, no. 4 (2022): 1444–1452, 10.1111/hex.13483. 35340091 PMC9327827

[34] E. Sellars , G. Pavarini , D. Michelson , C. Creswell , and M. Fazel , “Young People's Advisory Groups in Health Research: Scoping Review and Mapping of Practices,” Archives of Disease in Childhood 106, no. 7 (2021): 698–704, 10.1136/archdischild-2020-320452. 33208398

[35] R. K. J. Tan , W. Shan , E. Hummel , et al., “Youth Engagement and Social Innovation in Health in Low‐and‐Middle‐Income Countries: Analysis of a Global Youth Crowdsourcing Open Call,” PLOS Global Public Health 4, no. 7 (2024): e0003394, 10.1371/journal.pgph.0003394. 39024302 PMC11257312

[36] V. O. Femi‐Lawal , O. E. Ogunlana , T. Aderemi , et al., “Fostering Innovation and Building Youth Capacity in Global Health: Design, Implementation, and Evaluation of a Youth‐Led Digital Health Hackathon,” BMC Medical Education [Electronic Resource] 26, no. 1 (2026): 277, 10.1186/s12909-026-08633-w. 41566446 PMC12908292

[37] A. S. Laar , M. L. Harris , M. N. Khan , and D. Loxton , “Views and Experiences of Young People on Using mHealth Platforms for Sexual and Reproductive Health Services in Rural Low‐and Middle‐Income Countries: A Qualitative Systematic Review,” PLOS Digital Health 3, no. 12 (2024): e0000362, 10.1371/journal.pdig.0000362. 39630642 PMC11616881

[38] InciSio NUKC , “Global Health Education in Medical Schools (GHEMS): A National, Collaborative Study of Medical Curricula,” BMC Medical Education [Electronic Resource] 20, no. 1 (2020): 389, 10.1186/s12909-020-02315-x. 33115465 PMC7594419

[39] S. Malik , A. Alkoronky , M. Elmahi , et al., “Global Health in Undergraduate Education: Knowledge, Attitude, and Practice of Sudanese Medical Students Towards Global Health Education: A Cross‐Sectional Study,” BMC Medical Education [Electronic Resource] 23, no. 1 (2023): 230, 10.1186/s12909-023-04168-6. 37041577 PMC10091656

[40] A. Artaman , H. T. Bekele , and R. Millar , “Strengthening Global Health Education in the UAE: Assessing the Current Landscape and Future Directions,” Frontiers in Public Health 13 (2025): 1584880, 10.3389/fpubh.2025.1584880. 40575104 PMC12198231

[41] S. C. Garbern , G. Hyuha , C. González Marqués , et al., “Authorship Representation in Global Emergency Medicine: A Bibliometric Analysis From 2016 to 2020,” BMJ Global Health 7, no. 6 (2022): e009538, 10.1136/bmjgh-2022-009538. PMC9237874 35760436

[42] T. Adam , A. H. Ralaidovy , A. L. Ross , J. C. Reeder , and S. Swaminathan , “Tracking Global Resources and Capacity for Health Research: Time to Reassess Strategies and Investment Decisions,” Health Research Policy and Systems 21, no. 1 (2023): 93, 10.1186/s12961-023-00979-7. 37697313 PMC10496241

[43] D. C. Rodríguez , N. S. Jessani , and J. Zunt , “Experiential Learning and Mentorship in Global Health Leadership Programs: Capturing Lessons From Across the Globe,” Annals of Global Health 87, no. 1 (2021): 61, 10.5334/aogh.3194. 34307064 PMC8284496

[44] Y. A. Adebisi , N. D. Jimoh , and A. E. Bassey , “Harnessing the Potential of African Youth for Transforming Health Research in Africa,” Global Health 20, no. 1 (2024): 35, 10.1186/s12992-024-01039-7. 38664751 PMC11046890

